# Amputation of a type II diabetic patient with cutaneous leishmaniasis due to *Leishmania major*

**DOI:** 10.1186/s12879-021-06909-8

**Published:** 2021-12-07

**Authors:** Arezki Izri, Amina Bendjaballah-Laliam, Denis Sereno, Ihcene Kherachi Djenad, Zoubir Harrat, Mohammad Akhoundi

**Affiliations:** 1 Parasitology-Mycology Department, Avicenne Hospital, AP-HP, 125, Route de Stalingrad, Bobigny Cedex, 93009 France; 2 Unité des Virus Émergents (UVE: Aix-Marseille Univ-IRD 190-Inserm 1207-IHU Méditerranée Infection), Marseille, France; 3 Etablissement Public Hospitalier de Hadjout, Tipaza, Algérie; 4 Institut de Recherche Pour le Développement, Montpellier University, MIVEGEC, 34032 Montpellier, France; 5 Institut de Recherche Pour le Développement, Montpellier University, InterTryp, 34032 Montpellier, France; 6 Laboratoire d’Eco-épidemiologie Parasitaire et Génétique des Populations, Institute Pasteur of Algeria, Route du Petit Staoueli Dely Brahim, Algiers, Algeria

**Keywords:** Zoonotic cutaneous leishmaniasis, *Leishmania major*, Diabetes mellitus, Amputation

## Abstract

**Background:**

Leishmaniases are neglected tropical diseases of public health concern in Algeria. The immunocompromised patients with HIV, autoimmune diseases, or chronic alcohol abuse are at a higher risk of leishmaniasis. Herein, we present the case of an immunocompetent diabetic patient infected by *Leishmania major*, leading to life-threatening consequences.

**Case presentation:**

An Algerian diabetic patient developed a cutaneous lesion with large polymorphous inflamed granuloma and pyoderma gangrenosum in the left foot, following *L. major* infection. A delayed follow-up led to a treatment failure, resulting in the amputation.

**Conclusions:**

This report highlights the absence of timely treatment of *Leishmania* infection as a life-threatening point among high-risk diabetic patients. Clinicians should be aware of this parasitosis leading to severe complications in diabetic patients.

## Background

Leishmaniases are vector-borne diseases caused by obligate protozoan parasites from the *Leishmania* genus (Trypanosomatida: Trypanosomatidae). They are transmitted by the bite of infected female sandflies where the vertebrates like canids, rodents, bats, and hyraxes are reservoirs. Up to the present, 54 *Leishmania* species are known, with at least 21 human pathogenic ones [1].

Visceral (VL), cutaneous (CL), diffuse cutaneous (DCL), mucocutaneous (MCL), mucosal (ML), and post-kala-azar dermal leishmaniasis (PKDL) are clinical forms following *Leishmania* infections [2]. Ten countries (Afghanistan, Algeria, Colombia, Brazil, Iran, Syria, Ethiopia, North Sudan, Costa Rica, and Peru) encompass 75% of global CL incidence. Visceral leishmaniasis is highly endemic in six countries: India, Bangladesh, Sudan, South Sudan, Brazil, and Ethiopia [3]. In Algeria, a gradual increase in CL has been recorded over 30 years and ranks it among the most affected countries, with over 20,000 cases every year, making leishmaniasis a significant public health concern [3, 4]. Zoonotic cutaneous leishmaniasis (ZCL), caused by *L. major*, is prevalent in arid and semi-arid areas over the North Saharan fringe. Anthroponotic cutaneous leishmaniasis (ACL), caused by *L. killicki* (syn: *L. tropica*), is restricted to some foci located mainly in Constantine, Annaba, Ghardaia, and Tipaza [5]. Visceral leishmaniasis (VL), caused by *L. infantum*, is prevalent all over the coastal regions in northwestern Algeria (Oran, Tlemcen), in the Algerian Tell (Tizi-Ouzou, Bouira, Bord Menail, Tipaza, Blida, and Algiers), and the extreme south of the country (Hoggar) [6, 7].

## Case presentation

**Fig. 1:**
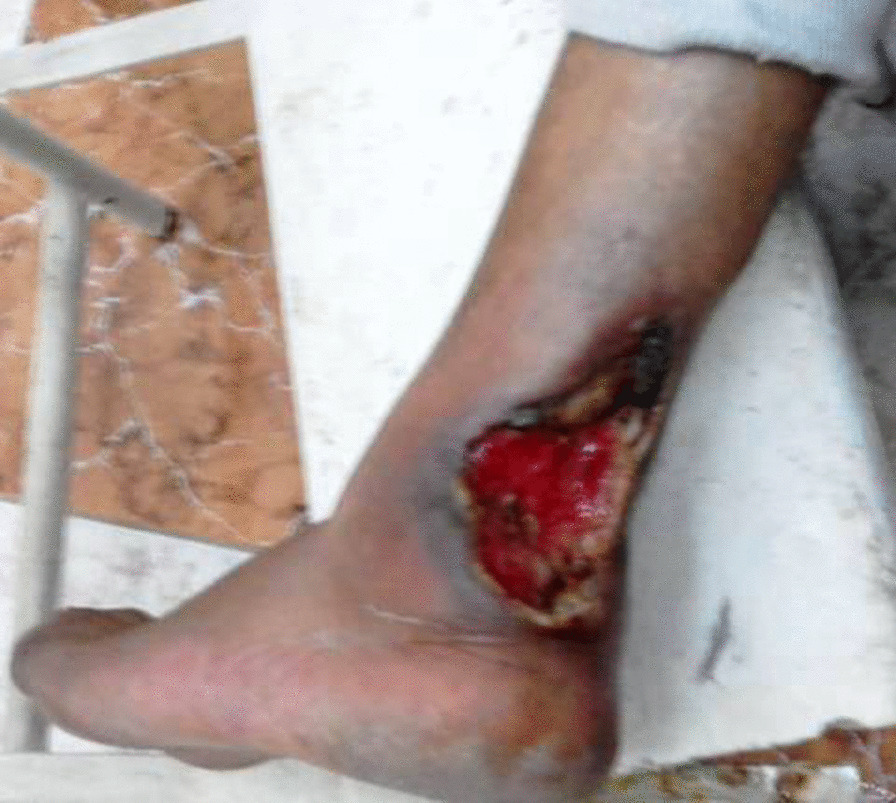
Extended ulcerative lesion with raised necrotic borders deforming upper part of left foot heel in an Algerian diabetic patient infected by *L. major*

A 49-year-old man was referred in March 2018 to Hadjout public hospital (80 km west of Algiers, Algeria) for a suspected cutaneous fungal infection. He was a primary school guardian who lived in a village in Chlef, 200 km far from Algiers. The primitive clinical examinations revealed a brownish ulcer, 10 cm diameter, and 0.3 cm depth, with a congestive necrotic border under epidermis on the left heel’s posterior surface, progressed during 10 months (Fig. 1). The patient was diagnosed diabetic one year ago and suffered from white coat syndrome without regular medical follow-up. There was no notion of maternally inherited disorders or injury due to unexplained causes. Additionally, no notion of smoking, obesity, or AIDS was recorded for this patient. Skin examination revealed the presence of papules with a swelling in the left foot. According to the patient, the cutaneous lesion was initiated in June 2017 by a small insect-bite-like red spot.

Blood analysis revealed a high glycated hemoglobin level (7.8%, compared to 4–6% of normal hemoglobin). The histopathological examination showed deep ulceration of the left foot with a large polymorphous inflamed granuloma compatible with pyoderma gangrenosum. No structures suggestive of *Leishmania* were observed in the histopathological examination. A bone demineralization with multiple small lacunar images involving the foot bones with an infectious origin was reported by radiography. There was necrosis in the ankle’s posterior soft parts with no notion of joint damage.

Three swabs were prepared from the patient’s lesion for mycological, bacteriological, and parasitological examinations. An etiological investigation revealed no fungal infection after direct examination or culture. The bacteriological examination demonstrated bacilliform bacteria, further identified as *Klebsiella pneumoniae* in differential media. Initial parasitological examination of the Giemsa-stained smear prepared from the patient’s lesion revealed no *Leishmania* infection by microscopy. On the contrary, DNA extraction of the Giemsa-stained smear and bidirectional sequencing of the ITS1-rDNA fragment amplification using conventional PCR [8], revealed the infection by *L. major*. The sequence was deposited in GenBank under the accession number XN348136.

The patient underwent an initial antibiotic therapy by oral pyostacine (3 g/day) for 10 days, leading to the elimination of bacterial infection one month post-treatment. Regrettably, the patient left the treatment incomplete for 5 months without receiving specific leishmaniasis treatment. This delay led to unfavorable CL lesion evolution and advanced infection culminating in extended wet gangrene, worsened over the time with no other treatment option, necessitating an above-the-knee amputation of his left leg in September 2018.

## Discussion and conclusions

Cutaneous leishmaniasis is a chronic disease in which the cutaneous lesion usually heals spontaneously, even without treatment [9]. The lesions vary in severity (e.g., lesion size), numbers, clinical appearance (e.g., dry or wet lesion), and spontaneous healing duration. The initial clinical manifestation is a slightly itchy red papule on the exposed areas, gradually extending to the surface and infiltrating in-depth, leading to central crusty ulceration, producing a typical lesion called oriental lesion [10]. Several pathogenic and non-pathogenic agents may be the cause of or contribute towards the progression of cutaneous lesions. Diabetes mellitus is a metabolic disease causing a deceptive ulcer in extremes, particularly in progressive stages. The feet of diabetic patients are at risk of developing a broad spectrum of clinical conditions, resulting in several diabetes-related complications. *Leishmania* parasite is one of the infectious etiologies evoked in a chronic leg ulcer. In case of misdiagnosis or failure in treatment, this chronic ulcer can progress and may lead to life-threatening consequences, like an amputation.

Most unusual and atypical clinical aspects of *Leishmania* infection are reported in immunodeficient patients [11]. Patients with advanced age, long history of diabetes mellitus, and cutaneous affection by pathogenic agents like *Leishmania*, bacterial or fungal microorganisms present a higher risk of amputation [12]. All mentioned criteria were present in our immunocompetent but high-risk patient. Chlef is a known endemic focus of ZVL (zoonotic visceral leishmaniasis) in Algeria, with *L. infantum* as the causative agent [13]. Unlikely, our patient was infected by *L. major*. Literature review depicts that atypical cutaneous presentation is observed in diabetic *Leishmania-*infected patients, and in very few cases, amputation is documented [14, 15]. The coexistence of *Leishmania* infection in diabetic patients favors the development of severe and extensive bacterial or fungal infections in lower limbs that may lead to amputation in inadequately treated cases.

To reduce diabetic-related mortality and morbidity, it is essential combining diabetes control with lesion treatment using antimicrobial medications that might include anti-leishmanial agents, particularly in patients living in leishmaniasis*-*endemic areas. The treatment of old-world CL is based on peri/intralesional injections of meglumine antimoniate (glucantime®), systemic therapy by liposomal amphotericin B, or oral fluconazole, depending on the *Leishmania* species, number, topography, and extent of lesions [16]. In our patient’s case, specialized wound care accompanied by several high-dose antibiotic courses like pyostacine could not prevent the left foot tissue’s deterioration, associated with a treatment failure. The latter can be attributed to several factors, including (i) long history of a diabetic trophic disorder responsible for gangrene of left foot, (ii) a superinfection with *L. major*, probably acquired by the bite of an infected sand fly (vector of *Leishmania* parasites), which have worsened the lesion, (iii) abandoning the treatment with glucantime, the first-line treatment for CL, for 5 months, (iv) an advanced age with a white coat syndrome (the patient suffered from stress and anxiety when encountering a physician, which caused him to refuse following the treatment process), (v) remoteness of the patient’s residence from the urban health center, and lack of timely access to proper health care, and (vi) a failure in treatment, due to delayed follow-up, which has led to the amputation of the left foot above the knee. This report highlights the absence of timely treatment of *Leishmania* infection as a life-threatening point among high-risk individuals (e.g., diabetes, AIDS).

## Data Availability

Not applicable.

## Abbreviations

HIV: Human immunodeficiency viruses
AIDS: Acquired immunodeficiency syndrome
